# A guided internet-delivered mindfulness-based cognitive therapy for insomnia: study protocol for a randomized controlled study

**DOI:** 10.3389/fpsyt.2025.1506183

**Published:** 2025-05-27

**Authors:** Zixuan Zeng, Fei Liu, Chenyi Zhu, Xiangyun Long, Jiahui Jiang, Lei Huang, Zheng Lu

**Affiliations:** ^1^ Department of Psychiatry, Tongji Hospital, School of Medicine, Tongji University, Shanghai, China; ^2^ Department of Psychiatry, The Affiliated Brain Hospital of Guangzhou Medical University, Guangzhou, China; ^3^ Department of Public Health and Preventive Medicine, Tongji Hospital, School of Medicine, Tongji University, Shanghai, China

**Keywords:** insomnia, mindfulness, cognitive behavioral therapy, Internet-based intervention, randomized controlled trial

## Abstract

**Background:**

Chronic insomnia disorder (CID) is highly prevalent and impacts individuals’ physiological, psychological and social functions. Although cognitive behavioral therapy for insomnia (CBT-I) is the recommended first-line treatment option for CID, approximately 20% of patients still exhibit limited effectiveness. The literature has shown that mindfulness can benefit CBT-I protocols and that mindfulness-based cognitive therapy (MBCT) is effective in treating insomnia. However, the effectiveness of therapist-guided internet-based MBCT-I (iMBCT-I) has not yet been tested.

**Objective:**

This study protocol aims to evaluate the effect of therapist-guided iMBCT-I on chronic insomnia among Chinese adults.

**Methods:**

This will be a two-armed, parallel group, randomized controlled study with 80 CID patients. The participants will be randomized to either the iMBCT-I program or the sleep hygiene education (SHE) program at an allocation ratio of 1:1 via simple randomization. Assessments will be carried out at baseline, at the end of the intervention (week 8) and at the follow-up time (week 20). The primary outcome was insomnia severity at week 8. Statistical analyses will follow the intention-to-treat (ITT) principle.

**Discussion:**

This is the first study to investigate the effectiveness of therapist-guided iMBCT-I in CID patients. Should it be effective, this study will provide evidence for clinical practitioners, therapists and patients to consider a new psychotherapeutic option and for technicians to perform self-guided iMBCT-I applications in the future.

**Clinical trial registration:**

https://www.chictr.org.cn, identifier ChiCTR240008430.

## Introduction

Insomnia, which primarily comprises sleep-initiating/maintaining difficulty or early morning awakening, is the most common sleep disorder. Approximately one-third of adults worldwide are estimated to experience symptoms of insomnia, with 5% to 10% meeting the diagnostic criteria for chronic insomnia disorder (CID) (1). In China, the prevalence of insomnia in the general population is 15.0%, as shown in a meta-analysis conducted in 2017 (2). A nationwide online survey conducted during the COVID-19 period reported a staggering 29.2% prevalence among the Chinese public (3). Numerous studies have shown that CID significantly impacts an individual’s physiological, psychological and social functions. It is associated with many adverse health outcomes, such as depression, anxiety, alcohol abuse and cardiovascular diseases, and may lead to various social dysfunctions, including reduced work efficiency, increased absenteeism, impaired social interaction and diminished quality of life (4–7).

The treatment of CID mainly includes pharmacological and nonpharmacological approaches, among which cognitive behavioral therapy for insomnia (CBT-I) is the most recommended first-line treatment option because of its proven efficacy, safety and long-lasting benefits (6). CBT-I is a multicomponent psychotherapy for insomnia that typically involves sleep hygiene education, relaxation response training, stimulus control, sleep restriction and cognitive therapy. It improves sleep quality mainly through restructuring maladaptive cognitions about sleep and promoting healthy sleep habits (8). However, adherence to CBT-I is often suboptimal. A meta-analysis indicated that the average dropout rate of self-help CBT-I at immediate post-treatment was 14.5%, which was not significantly different from the 16.7% observed in therapist-administered CBT-I (9). Furthermore, a large-scale randomized controlled trial of digital CBT-I (n=1721) showed that attendance rates gradually declined as sessions progressed, dropping to as low as 46% in the later stages (10). Besides, approximately 20% of CID patients still exhibit limited responsiveness to CBT-I or experience short-term efficacy, with a tendency for relapse (11). This may be because CBT-I mainly targets the correction of patients’ irrational sleep beliefs, while it intervenes less in their emotional regulation, control and nocturnal awakening (12). Therefore, the efficacy of CBT-I may be suboptimal for those with high levels of sleep-related arousal, increased negative emotions and reduced subjective well-being (13). Therefore, there is a need to include different elements of CBT-I as supplements.

Mindfulness is defined as “the awareness that emerges through paying attention on purpose, in the present moment, and nonjudgmentally to things as they are” (14). Mindfulness-based interventions (MBIs) are considered promising treatments for many health problems, including insomnia (15). Although MBIs are not typically listed as standard components of CBT-I, they could benefit CBT-I protocols by targeting experiential awareness, attention control and acceptance to address the cognitive and behavioral issues underlying poor sleep (16). By increasing awareness and disrupting negative thought patterns, it reduces rumination and arousal to improve sleep quality (17, 18). It also teaches participants to integrate mindfulness practices into their daily lives, which may lead to sustained long-term improvements in insomnia directly or indirectly (19). Mindfulness-based cognitive therapy (MBCT) is a systematic method of psychotherapy developed in the 1990s by Segal et al., who integrated mindfulness-based stress reduction (MBSR) with CBT (20), which was initially used for preventing depression relapse (21). Mindfulness-based cognitive therapy for insomnia (MBCT-I) is an integration of mindfulness and CBT-I and has been proven to be effective in treating insomnia. In 2017, Wong SY et al. adapted MBCT by replacing the content related to depression with insomnia-specific content and conducted a large-scale randomized controlled trial to prove the significant efficacy of MBCT-I in patients with CID (22). However, the MBCT-I was face-to-face and high-intensity, and only 42% of the participants completed at least six of the eight sessions. The high cost, long duration, and low adherence rates reflect common challenges in the current landscape of in-person psychotherapy (23). In addition, MBCT-I has also been proven to be effective in adolescents (24), older adults (25), breast cancer survivors (26) and patients with depressive or anxiety disorders (27, 28).

Nevertheless, the large and widespread population suffering from insomnia and limited mental health resources have hindered the adoption of traditional face-to-face psychotherapies among CID patients in China (29). Although CBT-I has been widely acknowledged as a golden approach for treating CID, obstacles such as a lack of skilled professionals, lengthy treatment cycles and high cost have limited the promotion of CBT-I in clinical implementation. Fewer than 1% of CID patients gained access to CBT-I (30). To address these challenges, internet-based CBT-I (iCBT-I) has emerged and garnered significant attention in recent years. Several recent meta-analyses have shown that therapist-guided iCBT-I not only yields outcomes comparable to those of face-to-face CBT-I for CID but also alleviates the economic burden for patients and saves medical resources (31–34). Therefore, therapist-guided iMBCT-I is expected to be a promising treatment approach for CID patients with high efficacy and accessibility. However, the effectiveness of guided iMBCT-I has not yet been tested in CID patients.

This is one of the earliest randomized controlled studies to evaluate the effectiveness of therapist-guided iMBCT-I in CID patients by comparing it to that of sleep hygiene education (SHE). In this study protocol, we will simplify the standardized MBCT format, reducing the session duration from 2.5 hours to a more manageable 1.5 hours, which may enhance the compliance and engagement of participants. The iMBCT-I program is not only tailored to the context of China but also more accessible for individuals seeking online interventions. If proven effective, this study will provide valuable evidence for clinical practitioners, therapists, and patients considering a novel psychotherapeutic option and offers insights for technicians involved in the future implementation of self-guided iMBCT-I applications.

The primary outcome is insomnia severity. The secondary outcomes include sleep quality; sleep parameters recorded via a sleep diary and actigraphy; the level of mindfulness; dysfunctional beliefs about sleep; anxiety; depression; quality of life; and attitudes towards traditional psychotherapy and online psychotherapy. Data about intervention satisfaction and suggestions will be collected.

The primary hypothesis for this trial is that the iMBCT-I will reduce insomnia severity by the end of the treatment (8 weeks).

The secondary hypotheses for this trial are as follows:

1. The iMBCT-I will improve sleep quality, level of mindfulness, quality of life and attitudes towards traditional psychotherapy and online psychotherapy by the end of the intervention (week 8).
2. The iMBCT-I will decrease dysfunctional beliefs about sleep, anxiety and depression by the end of the intervention (week 8).
3. The effect of the iMBCT-I will be maintained at the scheduled follow-up assessment (week 20).
4. Mindfulness levels, attitudes towards traditional psychotherapy or online psychotherapy at baseline will have an influence on both the short-term (week 8) and long-term (week 20) effects of the iMBCT-I.

## Methods

### Trial design

**Figure 1:**
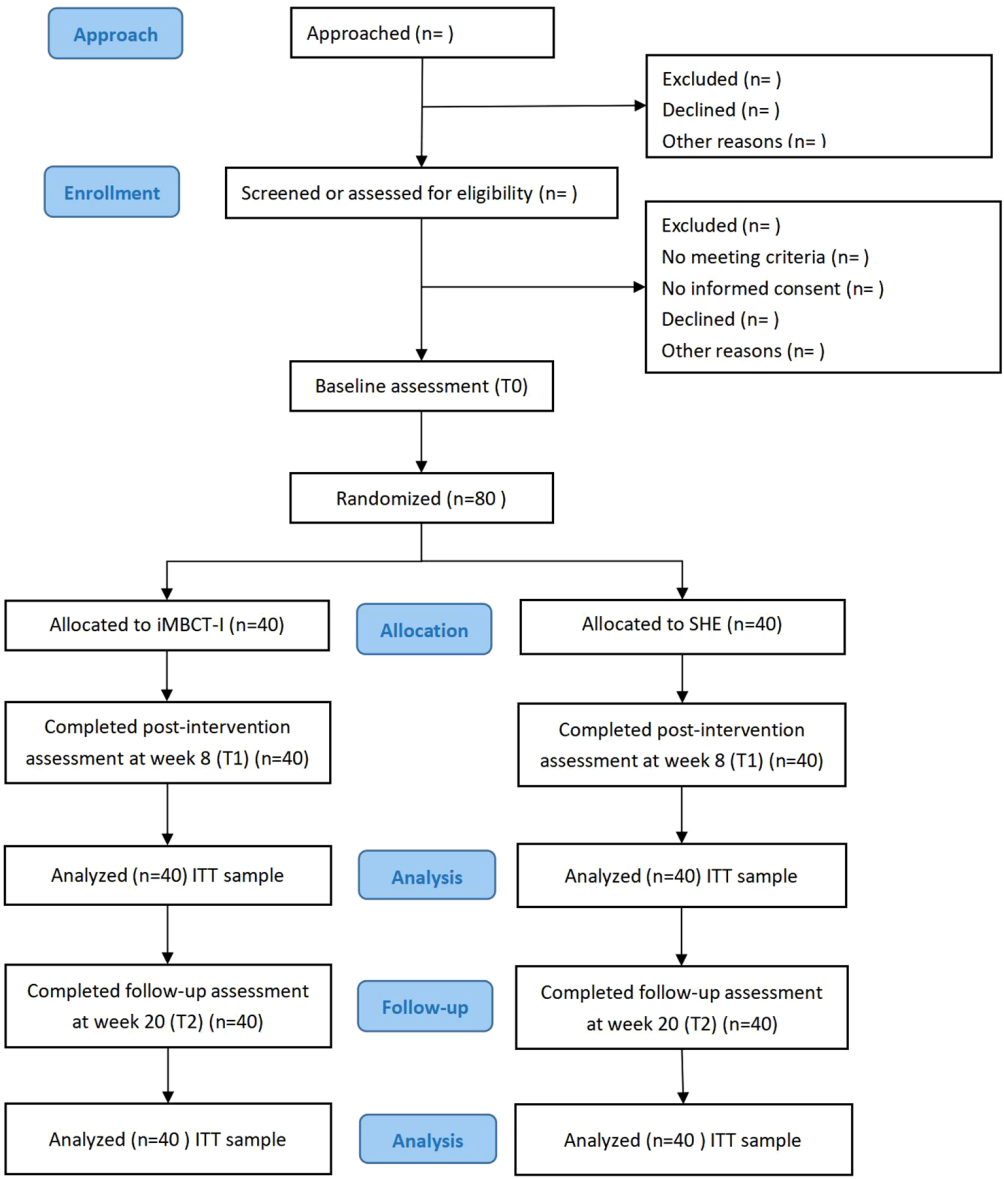
Participant flow chart with the targeted sample size.

This is a prospective, parallel-group, exploratory randomized (1:1 allocation ratio) controlled study. This study will be carried out following the CONSORT 2010 statement, as shown in **Figure 1**. Initially, interested participants will undergo preliminary screening via an online questionnaire. Those meeting the criteria will be further evaluated via video calls by two psychiatrists to determine eligibility for inclusion. All eligible participants will subsequently electronically sign informed consent forms before baseline assessment and intervention. After the intervention concludes, participants will be required to complete two assessments: one immediately post-intervention (8 weeks) and another three months thereafter (20 weeks). The signing of informed consent and all questionnaire assessments will be completed on Questionnaire Star (a popular online survey platform in China), and the intervention programs will take place at Tencent Meeting (a widely used online conferencing app in China).

This study protocol follows the SPIRIT 2013 checklist for clinical trial protocols, has received ethical approval from the Tongji Hospital of Tongji University Institutional Review Board (reference number: 2024–013) and has been registered with the Chinese Clinical Trial Registry (Registration Number: ChiCTR2400084300) (35). The SPIRIT checklist is displayed in **Supplementary Table S1**. The SPIRIT figure is displayed in **Supplementary Table S2**.

### Study setting

This study will be conducted in Tongji Hospital of School of Medicine, Tongji University, Shanghai, China. We plan to recruit participants through outpatient departments (e.g., psychiatry, neurology and general practice).

### Eligibility criteria

To be included, participants must fulfil the following criteria (1): Aged between 18 and 65 years with normal speech and cognitive functions, defined as a total score of >25 on the Montreal Cognitive Assessment (MoCA) (36) (2); having chronic insomnia disorder based on Insomnia Severity Index (ISI) >14 and DSM-5 diagnostic criteria that include (a) difficulties initiating and/or maintaining sleep, defined as sleep onset latency (SOL) and/or wake time after sleep onset (WASO) greater than 30 minutes, or early-morning awakening with inability to return to sleep; (b) sleep difficulty being present at least 3 nights per week and for a period of at least 3 months; (c) severe impairment of daytime functioning (3); being willing to complete the online MBCT-I or SHE program (4); agree to maintain the dosage and frequency of sleep medication throughout the study or discontinue usage for at least 2 weeks (washout period) prior to study enrollment among those who use sleep medication; and (5) voluntarily signing the informed consent form.

The exclusion criteria are (1): those who might be unable to use Tencent Meeting or complete sleep diaries and questionnaire assessments because of illiteracy or other reasons (2); life-threatening conditions (e.g., suicidal ideation, advanced cancer) or expected to experience circumstances that may severely affect compliance (e.g., pregnancy, dementia, substance abuse) (3); the presence of progressive medical illnesses related to the onset and/or course of insomnia (4); previously diagnosed with or currently suspected of having schizophrenia, major depressive disorders, bipolar disorders, anxiety disorders, eating disorders or substance abuse according to the Mini International Neuropsychiatric Interview (MINI) provided by two psychiatrists (5); having conditions that may worsen due to sleep restriction therapy, including uncontrolled hypertension, diabetes, seizure disorders, recent surgery, or engaged in transportation-related occupations (6); having night shift work or other work/study tasks that strongly affect the sleep rhythm (7); planning or having already received CBT, MBCT or other MBIs; and (8) self-reporting other sleep disorders (e.g., sleep apnea, restless leg syndrome, rapid eye movement sleep behavior disorder (RBD) or hypersomnia) or screening positive for sleep apnea (STOP-Bang score ≥ 3) (37), restless leg syndrome (meeting International Restless Legs Syndrome Study Group,IRLSSG criteria) (38), RBD (answering “yes” in REM Sleep Behavior Disorder Single-Question Screen, RBD1Q) (39), or hypersomnia (Epworth Sleepiness Scale score > 10) (40).

## Interventions

### Intervention description

#### Intervention group: iMBCT-I

Participants will receive a 12-hour iMBCT-I treatment. MBCT-I consists of weekly 1.5-h sessions over 8 weeks online through the Tencent Meeting, with mindfulness practice as homework six times a week.

The iMBCT-I program was adapted from the original format of Segal et al., which targeted mainly depression and consisted of eight 2.5-hour sessions held on a weekly basis (20). The modification mainly followed the MBCT-I in Wong SY and colleagues’ research, making it tailored for CID patients (22). Due to the limitation that Tencent Meeting displays a maximum of 25 members’ videos per page, we set the maximum group capacity to 20 individuals to ensure that all members can consistently maintain face-to-face interaction online.

The iMBCT-I program will be conducted by certified instructors with over 2 years of experience in teaching MBCT. Prior to the treatment, an initial orientation session will be held to clarify the objectives and tasks for the 8-week duration and to facilitate mutual acquaintance among group members. Thereafter, the weekly sessions will be conducted according to the respective theme. Themes of iMBCT-I are Awareness and Automatic Pilot (week 1), Recognizing the Dysfunctional Beliefs in Insomnia (week 2), Thoughts Are Not Facts (week 3), A New Perspective (week 4), Awareness is the Start of Change (week 5), Allowing/Letting Be (week 6), “How Can I Best Take Care of Myself?” (week 7) and Maintaining and Extending New Learning (week 8). The program includes sequential instruction in mindfulness practices such as body scan, mindful eating (raisin exercise), pleasant/unpleasant events calendar, sitting meditation, “seeing” or “hearing” exercise, 3-minute breathing space, mindful stretching, and walking. In addition, the iMBCT-I program includes both cognitive and behavioral elements. The cognitive component of iMBCT-I primarily includes psycho-education related to sleep hygiene, covering the nature of sleep and insomnia, which are factors influencing sleep and good/bad sleep habits. Simultaneously, participants are taught to identify ruminative or dysfunctional thoughts associated with insomnia, foster awareness of these thoughts, explore the accompanying feelings and emotions that emerge in response to insomnia, and approach all of them with an accepting and nonjudgmental mindset. Unlike CBT-I, the philosophy of iMBCT-I is to cultivate an accepting, nonjudgmental relationship with those beliefs, thoughts and emotions instead of challenging them. The behavioral components include sleep hygiene, sleep restriction and stimulus control, following the guidelines outlined in the CBT-I manual. Approximately 80% of the course time is dedicated to the mindfulness component, whereas the remaining 20% focuses on the CBT-I component.

#### Control group: SHE

Participants will receive a 1.5-hour initial sleep hygiene education session online through the Tencent Meeting. The online sleep hygiene education session will be carried out according to the Sleep Health Management Handbook recommended by the Chinese National Health Commission (41). It delivers standardized content covering insomnia symptoms and their impact, prevalence and underlying causes. In addition, it provides guidance on when to seek medical assistance and offers basic lifestyle, environmental, and behavioral strategies to improve sleep quality.

The choice of a one-time SHE session as the control condition aligns with prior studies investigating other cognitive-behavioral interventions for insomnia (10, 42). While SHE differs in contact time from the iMBCT-I program, it serves as an active control condition that provides standardized, evidence-based sleep health knowledge without including structured mindfulness or cognitive-behavioral techniques. This approach allows us to isolate the specific effects of iMBCT-I from general educational benefits while maintaining ecological validity.

Participants in the SHE group will be attention-matched with those in the iMBCT-I group to control for expectancy and engagement effects. First, both groups will receive standardized informed consent, which clearly states: “This study compares two different approaches to improving sleep. You will be randomly assigned to one of the following: Program A – improving sleep through iMBCT-I, or Program B – improving sleep through SHE. Both are evidence-based, but it is currently unclear which is more suitable for most people.” This framing is intended to equalize treatment expectancy across groups. Second, both interventions will be delivered in an online group format via Tencent Meeting. Third, to maintain comparable levels of engagement and attention, participants in the SHE group will be asked to keep sleep diaries, just as in the iMBCT-I group. Research assistants will send daily reminders via smartphone to facilitate compliance. Considering that the iMBCT-I group will receive seven additional weeks of structured intervention, the SHE group will be administered seven weekly follow-up questionnaires to maintain engagement parity. These questionnaires will include items such as “How has your sleep been over the past week?” and “Which aspects of the sleep hygiene education have you applied?” Fourth, both groups will receive the same incentive: a free actigraphy upon completion of the study. Finally, post-intervention satisfaction assessments will be conducted for both groups to evaluate their subjective experiences with the respective interventions.

### Criteria for discontinuing or modifying allocated interventions

If a participant withdraws informed consent or experiences a serious adverse event (such as self-harm or impulsive behaviors) during the study, they will be immediately withdrawn from the trial and provided with appropriate treatment as necessary.

### Relevant concomitant care permitted or prohibited during the trial

Participants are permitted to relevant concomitant care throughout the study.

### Outcomes

#### Primary outcome measures

In this study, the Insomnia Severity Index (ISI) will be used to measure the severity, nature and impact of insomnia (43). It consists of 7 items and is rated on a 5-point scale ranging from 0 (no problem) to 4 (very severe problem). A total score of 8–14 suggests subclinical insomnia, and a total score over 14 indicates clinical insomnia. A reduction in ISI scores of 8 or more points from baseline is regarded as a response to treatment, and an absolute score on the ISI of 7 points or less is regarded as remission (44, 45). The Chinese version of ISI was translated and validated by Yang et al. and its Cronbach’s α was 0.94 (46). And it is supported to be a valid outcome measure for CBT-I by literature (47).

#### Secondary outcome measures

At baseline, demographics (e.g. age, gender, marital status, educational background, income), related clinical data (e.g. use of medication) and descriptive data on lifestyle practices (e.g. smoking and alcohol use).

Sleep quality is assessed by the Pittsburgh Sleep Quality Index (PSQI). The PSQI is a self-rated questionnaire comprising 19 individual items that generate seven component scores: subjective sleep quality, sleep latency, sleep duration, habitual sleep efficiency, sleep disturbances, use of sleeping medication and daytime dysfunction (48). These components are then summed to a total score, which ranges from 0 to 21, with higher scores indicating poorer sleep quality. The Chinese version demonstrated high internal consistency, and its Cronbach’s α was 0.83 (49).

The Five Facet Mindfulness Questionnaire (FFMQ) is a 39-item instrument that measures five distinct facets of mindfulness, namely observing, describing, acting with awareness, non-judging, and non-reacting (50). It is scored using a 5-point Likert scale, ranging from 1 (never or very rarely true) to 5 (very often or always true). Those have greater mindfulness levels have higher total scores. The FFMQ has been translated into Chinese version and widely used in China. The Cronbach’s α of the Chinese version was 0.83 for the community sample and 0.80 for the clinical sample (51).

The Patient Health Questionnaire-9 (PHQ-9), a globally used tool to screen and measure the severity of depression, is applied to measure the depressive symptoms of CID patients in this study (52). It consists of 9 items, with a 4-point Likert scoring system, ranging from 0 (not at all) to 3 (nearly every day). The total score varies from 0 to 27. Scores of 5, 10, 15, and 20 represent mild, moderate, moderately severe, and severe depression, respectively (52). In Chinese population, the Cronbach’s α was 0.86 (53).

The Generalized Anxiety Disorder 7-item scale (GAD-7) is used to assess anxious levels by evaluating the frequency of being bothered by specific symptoms over the past two weeks (54). Each item is scored from 0 (not at all) to 3 (nearly every day). The total score ranges from 0 to 21. Scores of 5, 10, 15 represent mild, moderate and severe anxiety, respectively (54). GAD-7 has been validated among Chinese population, and the Cronbach’s α was 0.90 (55).

To assess health related quality of life, the SF-12 Health Survey is used, which contains 12 items and 2 subscales: physical component summary (PCS) and mental component summary (MCS) (56). Through standardized transformation models, these scores are converted into normalized scores ranging from 0 to 100, with higher scores suggesting better health related quality of life. The psychometric properties have been confirmed for Chinese population, with Cronbach’s α being 0.86 (57).

The Attitudes Toward Seeking Professional Psychological Help Scale-Short Form (ATSPPH-SF) is a revised 10-items instrument designed to assess attitudes toward psychotherapy (58). The scale comprises three dimensions: openness to seeking professional help, perceived effectiveness of professional help and preference for independent problem-solving. Each item is rated on a 4-point Likert scale (0 = strong disagreement and 3 = strong agreement), with some items reverse-scored. Higher total scores demonstrate more positive attitude toward psychotherapy. The ATSPPH-SF has been translated and validated among Chinese population, with Cronbach’s α values ranging from 0.71 to 0.82 for its subscales and total score (59). In order to collect the attitude towards online psychotherapy, an adapted version of ATSPPH-SF is applied, with the term “psychotherapy” being replaced by “online training” thoroughly (60).

To assess sleep-related cognition, the Dysfunctional Beliefs and Attitudes about Sleep Scale 16-item version (DBAS-16) is used (61). Items are scored on a 5-point Likert scale (1=strongly agree and 5= strongly disagree), with lower scores indicating severer dysfunctional beliefs and attitudes. The DBAS-16 comprises four subscales, namely consequences of insomnia, sleep worry, sleep expectations, and medication. The DBAS-16 has been validated among Chinese population, with a Cronbach’s α value of 0.80 (62).

Sleep parameters include sleep onset latency (SOL), wakefulness after sleep onset (WASO), total sleep time (TST), sleep efficiency (SE) defined as the ratio of TST to total time spent in bed (TIB) and sleep perception index (SPI). Data will be collected through subjective entries in the sleep diary and objectively recorded by actigraphy (HUAWEI Smartwatch). Actigraphy can provide relatively accurate sleep parameter data while allowing for at-home monitoring. Its advantages include cost-effectiveness, convenience, portability and higher adherence, making it widely used in both clinical research and practice (63). Additionally, the actigraphy will analyze sleep states, including deep sleep, light sleep and rapid eye movement (REM) sleep. According to the research carried in Beth Israel Deaconess Medical Center in Harvard Medical School, the sleep states analyzed in HUAWEI Smartwatch achieved an accuracy of 79-92%, when the Electrocardiogram based Cardiopulmonary Coupling analysis (ECG-based CPC) was employed as true-event reference (64). Participants were required to keep daily sleep dairies and provide the actigraphy data during a 2-week baseline period, during the 8-week treatment and for 2 weeks prior to the scheduled follow-up assessment at 20 weeks, respectively. Sleep diaries will be recorded via smartphone and research assistants will send daily reminders via the smartphone to encourage participants to complete their entries on time. SPI is estimated using total sleep time (in hours) as perceived by the participants (via sleep diaries) divided by the total sleep time (in hours) measured by actigraphy (65).

Other measures include the motivation, frequency, duration and preference time of mindfulness practice in the MBCT-I group which is recorded during treatment and follow-up. Once the treatment is completed, we will investigate the intervention satisfaction of participants in both groups on arrangement, homework, leaders, atmosphere, effectiveness and technical difficulties. Medication use including changes in medication will be monitored in baseline, week 8 and week 20.

#### Attendance and intervention completion

Retention rate (number and proportion that attended each class) of participants in MBCT-I group are documented. And reasons for non-attendance will be recorded. Participants who attended six or more iMBCT-I sessions will be considered as having completed the intervention. For those who did not complete the intervention, researchers would conduct interviews to inquire about the relevant reasons. In addition, the completion of the sleep diary, actigraphy usage, and the number of sessions attended are collected as indicators of adherence.

#### Safety assessments

We will record the possible adverse events in detail during the intervention period, including the time of occurrence, duration, and treatment measures of the adverse events. Common adverse events of iMBCT-I may include anxiety, emotional or physical discomfort, sleep disruption and daytime sleepiness, and these possible conditions will relieve or disappear as the intervention progresses. Serious adverse events will be reported to the Tongji Hospital of Tongji University Institutional Review Board in a timely manner.

### Sample size

With reference to the results of a large-scale randomized controlled trial conducted by Lee M. R. which compared the effects of online CBT-I with SHE on chronic insomnia patients, we estimated that the ISI score reductions in the iMBCT-I group and the SHE group were 7.7 and 3.1, respectively, with a common standard deviation of 5.5 (42). Assuming an alpha level of 0.05 and a power level at 0.9, the calculated minimum sample size was 30 participants per group. Estimating a dropout rate of 20%, we plan to recruit 80 participants in total.

## Assignment of interventions

Once recruitment is completed, eligible participants will be randomly allocated to either iMBCT-I group or SHE group with an allocation ratio of 1:1, using simple randomization facilitated by computer-generated random numbers in IBM SPSS 25.0 (66). The generated sequences will be placed in a sealed, opaque, identical-looking envelope, ensuring that the evaluator is unaware of the allocation. The study coordinator will assess baseline information and then inform the attending physician to refer participants to a research team. The study coordinator will not recruit participants.

## Blinding

This will be an open-labeled trial. Participants could not be blinded to treatment allocation as participants are notified of the two groups: iMBCT-I and SHE. Intervention leaders will be blinded to the group assignment and study hypotheses. A statistician who is not part of the research team will carry out the randomization and allocation procedure. The research team will have limited contact with the statistician to prevent allocation bias. The research assistant responsible for statistical analyses will be blinded to study hypotheses as well as randomization, assessment and intervention procedures.

## Data collection and management

Assessments will be conducted at week 0 (baseline), week 8 (post-intervention) and week 20 (3 months after intervention). Details are displayed in **Table 1**.

**Table 1:** Assessment schedule.

Outcome	Measure	Time of assessment			
		Screen	Baseline (week 0)	Post-intervention (week 8)	Follow-up (week 20)
Socio-demographics	SR	X			
CID	MINI	X			
Insomnia severity	ISI	X	X	X	X
Sleep quality	PSQI		X	X	X
Mindfulness level	FFMQ		X	X	X
Depression	PHQ-9		X	X	X
Anxiety	GAD-7		X	X	X
Quality of life	SF-12	X	X	X	X
Attitudes	ATSPPH-SF	X	X	X	X
Dysfunctional beliefs	DBAS-16		X	X	X
Sleep parameters	Actigraphy		X	X	X
Treatment adherence	Number of completed sessions			X	X

SR-self reported measure; MINI- Mini International Neuropsychiatric Interview (MINI) provided by two psychiatrists; ISI- Insomnia Severity Index; PSQI- Pittsburgh Sleep Quality Index; FFMQ- The Five Facet Mindfulness Questionnaire; PHQ-9- The Patient Health Questionnaire-9; GAD-7- The Generalized Anxiety Disorder 7-item scale; SF-12- The 12-item Short Form Health Survey; ATSPPH-SF- The Attitudes Toward Seeking Professional Psychological Help Scale-Short Form; DBAS-16- Dysfunctional Beliefs and Attitudes about Sleep Scale 16-item version.

### Plans to promote participant retention and complete follow-up

Participants will be fully explained the importance of completing assessment at baseline, post-intervention and follow-up during recruitment. Each participant will receive a notification on the smartphone to complete the assessments online within 2 days. All questions were mandatory to ensure no missing data, and participants may pause and report to the research assistant at any time. Phone reminders will be provided if assessments were not completed on time.

### Data management

Electronic Medical Record Form (ECRF) will be utilized to collect data. All data will be stored in a highly secured database to ensure safety. The data will be backed up periodically (every 4 weeks). Electronic signed consent forms will be stored in our online assessment platform. The printed version will be stored in the locked room in our department. The raw data and all steps taken in the analysis will be documented in ECRF and SPSS to enable future researchers to understand and reuse the data. All data will be retained until 10 years after the completion of the study. Participant information will be protected and participant anonymity will be ensured to protect the rights of each participant.

## Statistical methods

### Statistical methods for primary and secondary outcomes

The statistical analyses will follow the intention-to-treat (ITT) principle. Missing values will be handled using a last observation carried forward (LOCF) analysis. Descriptive statistics at baseline will be computed using frequencies and percentages for categorical variables, means with standard deviations (SD) or medians with lower and upper quartiles for continuous variables. Tests of statistical significance between groups will be made. Clinical remission rate of insomnia in week 8 and week 20 will be calculated. All analyses will be conducted using IBM SPSS 25.0. A two-tailed p-value < 0.05 will be considered statistically significant.

#### Primary outcome analysis

Changes between groups in ISI scores from baseline to week 8 will be evaluated using paired t-tests (for normally distributed data) or paired Mann-Whitney U tests (for non-normally distributed data). The between group effect sizes (Cohen’s d) will be calculated using the mean differences from baseline and the SDs of the two groups at week 8.

A Univariable linear regression will be conducted to evaluate the effect of MBCT-I on ISI score reduction. The change in ISI scores from baseline to week 8 will be used as the dependent variable, while the intervention group (MBCT-I vs. SHE) will be included as the independent categorical variable. The regression coefficient (B) will represent the estimated effect of MBCT-I on ISI score reduction.

#### Sensitivity analysis

To account for potential confounders, a multivariable linear regression analysis will be performed, adjusting for covariates including gender, age, marital status, educational background, and baseline ISI scores.

#### Secondary outcome analysis

Changes between groups in scores of PSQI, FFMQ, PHQ-9, GAD-7, SF-12, ATSPPH-SF and DBAS-16, insomnia remission rate and all sleep parameters values from baseline to week 8 will be evaluated using paired t-tests (for normally distributed data)/Mann-Whitney U tests (for non-normally distributed data). Mixed-effect models for repeated measures will be used to evaluate the effects of the online MBCT-I on scores of ISI, PSQI, FFMQ, PHQ-9, GAD-7, SF-12, ATSPPH-SF and DBAS-16 as well as all sleep parameters values over time (from baseline to week 20). The model will include treatment group, time, treatment-by-time interaction and baseline covariates.

#### Exploratory analysis

In order to identify predictors of effectiveness, subgroup analyses will be conducted to compare the efficacy of iMBCT-I among participants with different characteristics, such as demographics, mindfulness levels (FFMQ) and attitudes towards psychotherapy (ATSPPH-SF). Additionally, the relationship between the number of attended sessions and treatment efficacy will be explored using regression analysis, with session attendance as an independent variable and ISI score reduction as the dependent variable.

Structural equation models will be applied to identify possible mediators and moderators of intervention efficacy such as mindfulness levels (FFMQ) and attitudes towards psychotherapy (ATSPPH-SF) at baseline.

### Plans to give access to the full protocol, participant level-data and statistical code

The full protocol and statistical analysis plan are available on request to the corresponding author after the manuscript published according to the data dissemination guidelines.

## Oversight and monitoring

### Composition of the coordinating center and trial steering committee

This is a single-center study designed and implemented by the Tongji Hospital, School of Medicine, Tongji University. The steering committee is comprised of authors Prof. Lei Huang and Zixuan Zeng. The committee members participated in the study design and provided suggestions for the experiment. The data security monitoring committee is independent of the host organization, including statistician and Prof. Zheng Lu as the chairman. The supervision and management department can conduct audits to assess the clinical research behavior and compliance with the protocol, SOPs, GCP, and applicable regulatory requirements.

### Composition of the data monitoring committee, its role and reporting structure

This study do not have Data Security Monitoring Board (DSMB) because no serious adverse events were reported with MBCT-I in previous studies. Research physicians will be contacted as security officers within 24 h if an adverse event occurs. If the safety officer assesses that the adverse event is related to the intervention, relevant safety measures will be taken. Serious adverse events will be reported to Tongji Hospital of Tongji University Institutional Review Board.

### Adverse event reporting and harms

All adverse events reported by participants and observed by researchers, as well as causal assessments of adverse events following the intervention, will be recorded in the Case Report Form (CRF). MBCT-I is classified as a non-pharmacological treatment. To date, few instances have been identified that could potentially harm participants’ health (67, 68). The sleep restriction component (which involves reducing the time spent awake in bed to enhance sleep efficiency) may initially cause discomfort for participants. Other potential adverse reactions may include distress, anxiety, or exacerbation of insomnia during the psychotherapy process. All adverse events occurring during the study will be carefully monitored and documented to determine their relationship to the intervention.

### Frequency and plans for auditing trial conduct

The study will be supervised by the hospital safety supervision committee, which will look after the study design re-evaluation, procedure execution, medical ethics, participant safety, and data management. The research team will discuss protocol amendments and notify the PI. The PI will have a copy of the modified agreement. Any behavior deviating from the agreement will be recorded with the reporting form.

### Dissemination plans

The results of this study will be communicated in international conferences and be published in peer-reviewed journals. Both positive and negative results will be reported.

## Discussion

To the best of our knowledge, this study protocol is the first to investigate the feasibility and effectiveness of therapist-guided iMBCT-I compared with sleep hygiene education in patients with chronic insomnia disorders via a randomized controlled design. This study will also test the effectiveness of iMBCT-I on secondary outcomes such as the level of mindfulness, quality of life, symptoms of depression and anxiety, and dysfunctional beliefs about sleep. We anticipate that iMBCT-I will reduce insomnia severity and that this effect will be maintained at the follow-up assessment. We also anticipate improvements in the level of mindfulness, quality of life, symptoms of depression and anxiety and dysfunctional beliefs about sleep, as they are closely correlated with sleep quality (68–71). We believe that this study could fill the research gap in evidence regarding the effectiveness of MBCT for CID. We will provide a more cost-effective, accessible and lower-intensity online intervention.

This study protocol has some strengths. First, the intervention is innovative, integrating the benefits of both mindfulness and CBT-I while being delivered by trained therapists. The iMBCT-I program has been tailored to the Chinese context and adapted into more manageable 1.5-hour sessions to enhance participant compliance and engagement. Second, we will conduct a comprehensive assessment of participants’ sleep, utilizing both subjective (questionnaire, sleep diary) and objective (actigraphy) measures in many different dimensions (insomnia severity, sleep quality, sleep parameters, sleep states and sleep-related dysfunctional beliefs). All measurement tools in this study are used worldwide, with proven reliability and validity. In addition, exploratory analysis of factors influencing outcomes will provide insights into identifying subgroups of individuals who are better suited for receiving iMBCT-I, which will aid in guiding treatment choices for clinical practitioners and patients with CID.

This study protocol has several limitations. First, a double-blinded study design cannot be used in the current study. Another limitation is the structural difference between the intervention and control groups in terms of treatment duration and provider contact. While the iMBCT-I group undergoes an 8-week structured program with therapist-led sessions, the SHE group receives only a single-session educational intervention. This discrepancy may introduce non-specific effects, as greater provider interaction and prolonged engagement in the intervention group might contribute to improvements in sleep beyond the specific effects of MBCT-I. Future studies will consider using structurally equivalent iCBT-I and provider contact as a comparator to investigate whether iMBCT-I demonstrates superior efficacy.
